# Engineering the *Drosophila* Genome for Developmental Biology

**DOI:** 10.3390/jdb5040016

**Published:** 2017-12-11

**Authors:** Dagmara Korona, Stefan A. Koestler, Steven Russell

**Affiliations:** Department of Genetics, University of Cambridge, Downing Street, Cambridge CB2 3EH, UK; dk500@cam.ac.uk (D.K.); sak76@cam.ac.uk (S.A.K.)

**Keywords:** *Drosophila*, genome engineering, CRISPR-Cas9, protein tagging

## Abstract

The recent development of transposon and CRISPR-Cas9-based tools for manipulating the fly genome in vivo promises tremendous progress in our ability to study developmental processes. Tools for introducing tags into genes at their endogenous genomic loci facilitate imaging or biochemistry approaches at the cellular or subcellular levels. Similarly, the ability to make specific alterations to the genome sequence allows much more precise genetic control to address questions of gene function.

## 1. Introduction

It is over a century since Thomas Hunt Morgan and his students established *Drosophila melanogaster* as a powerful system for exploring the basis of heredity [1,2]. By the latter period of the 20th century, due in part to the pioneering work of Ed Lewis, along with the large scale genetics screens by Christiane Nüsslein-Volhard and Eric Wieschaus, and coupled with the tremendous progress in molecular biology, the fly became well established as a model for developmental biology [2]. With an increasingly sophisticated range of genetic tools, very efficient transgenesis and the well-described anatomy available, considerable progress was made in characterising conserved developmental pathways [3]. The advent of genome sequencing and the post-genomic era inevitably led to new approaches in developmental biology, in particular, functional analysis of genes identified in high-throughput genomics screens or fly orthologues of mammalian genes identified in such studies [4]. Despite its sophistication, the major drawback of the fly as an experimental model was the lack of an easy system for targeting genomic changes in the organism via the type of homologous recombination used in yeast or vertebrate cells. While the development of gene targeting approaches [5,6] by the Golic laboratory addressed this deficiency in part, the systems are cumbersome and success tends to be highly locus specific. More recently, a range of transposon-based methods has allowed much greater flexibility in precisely manipulating genes in situ [7,8], however, the very recent advent of Clustered, Regularly Interspaced, Short Palindromic Repeat (CRISPR)-based genome engineering now offers the prospect of making virtually any desired change to the genome sequence much easier than previously achievable. Together, these methods are opening new avenues in developmental biology research, facilitating precise questions about gene/protein function, allowing the targeted expression or knock out of any gene and enabling high-resolution imaging of proteins, often in live tissues. Here we overview the most recent methods for manipulating the fly genome with a focus on approaches tackling gene manipulation and in vivo localisation for developmental studies.

## 2. Classical Methods

One of the major landmarks in the progression of the fly as an experimental tool for the study of development was the establishment of a robust method for generating transgenic animals via the *P* element transposon [9]. Since its introduction, a range of sophisticated *P* element-based tools have been constructed; including the Gal4-*UAS* system for precise spatial and temporal control of gene expression [10]; enhancer trapping for the genome-wide identification of developmentally regulated genes [11,12] and methods for systematic gene misexpression with engineered *P* transposable elements (EP) [13]. The utility and use of these by now classic tools have been well described in many reviews, and we here focus on some of the more modern applications of transposons. While the *P* element has been the workhorse of *Drosophila* transgenesis for over 30 years, it does exhibit an insertion bias [14], particularly towards gene promoters, making it less useful for targeting other genic regions such as introns. The establishment of other transposon systems, particularly *Minos* and *piggyBac* [15,16], along with the site-specific integrase PhiC31 [17], have opened more of the fly genome to engineering approaches. Considerable detail on newer engineering methods have recently been well reviewed by Venken and colleagues [18]; here we briefly touch on the most important systems, with a focus on those facilitating imaging studies or introducing precise changes to the genome sequence.

The introduction of a variety of inducible site-specific recombination systems, principally Flp-*FRT* [19] and Cre-*Lox* [20], have enhanced our ability to manipulate gene expression in space and time or to precisely engineer the genome. For example, introducing transgenic constructs containing *FRT* sites into the genome has considerable utility for functional and imaging analyses, facilitating spatial or temporal control of gene activation [21]. Generating mitotic or germ-line clones, techniques that are necessary to overcome pleiotropic or maternal effects of mutations and study gene functions during later stages of development, are simplified by the use of chromosomes with *FRT* sites close to the centromere. Similarly, the use of Flp-mediated recombination sees widespread use in methods aimed at marking specific populations of cells for lineage tracing or functional studies, particularly in the nervous system, and these have recently been well reviewed by others [22,23,24,25,26,27]. 

**Figure 1:**
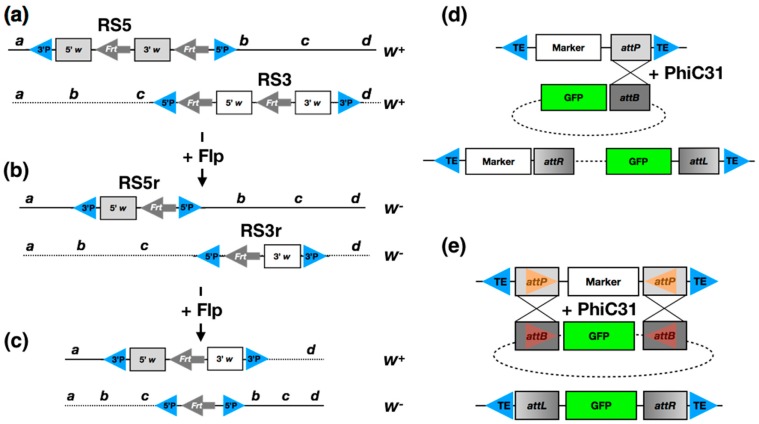
**Genome engineering.** (**a**–**c**) **The Golic method for generating precise chromosomal deletions.** (**a**) Two *P* elements, designated RS3 and RS5, are inserted at different locations (designated by a–d) on two homologous chromosomes and kept in separate fly lines. The elements contain a functional mini-*white* gene composed of multiple exons that for simplicity are drawn as grey or white boxes, representing 5′ and 3′ portions of the gene. There are two *Frt* sites (grey arrows) in each element, one of which is located within a mini-*white* exon. The elements differ in the position of the second *Frt* site and the orientation of the construct with respect to the *P* element ends (blue triangles). (**b**) Internal Flp-driven recombination between the *Frt* sites produces remnant forms of the *white* genes such that RS5r contains the 5′ end and RS3r the 3′ end with the intronic *FRT* site remaining. Each of these remnant elements are generated in separate fly lines that are phenotypically white eyed. (**c**) RS5r and RS3r elements are brought together in trans in a fly along with a source of Flp recombinase. FLP-mediated recombination between the elements produces a reconstituted functional *white* gene and the intervening genomic DNA is deleted. The reciprocal event creates a tandem duplication of the deleted segment, separated by an *FRT* site, but no white gene. (**d**) **The PhiC31 system.** A transposon (blue triangles mark the transposon ends) carrying a marker gene for genetic tracking and an *attP* site is inserted into the genome. Providing an *attB* containing plasmid with a gene or sequence of interest, in this case GFP, and a source of PhC31 integrase results in high efficiency integration of the plasmid into the genomic location. (**e**) **Recombinase-mediated cassette exchange (RMCE).** A transposon (blue triangles mark the transposon ends) carrying a marker gene flanked by *attP* site is inserted into the genome. Providing a plasmid with a gene or sequence of interest, in this case GFP, flanked by *attB* sites and a source of PhC31 recombinase results in high efficiency replacement of the genomic marker with the sequence of interest. With RMCE the inverted orientation of the *attP* and *attB* sites is critical for producing the desired exchange.

Projects by Exelixis and the DrosDel consortium have generated several thousand insertions with transposons carrying *FRT* sites that allow precise chromosome engineering [28,29]. For example, using the approach developed by Golic and Golic [30] (Figure 1a–c), DrosDel generated over 3000 insertions of re-arrangement screen 5 and 3 (RS5 and RS3) *P* elements, using these to build deletion coverage for almost 80% of the genome [29]. More importantly, combining the Exelixis and the DrosDel collections allows the construction of over 500,000 deletions ranging in size from 1 bp to 1 Mb that are precisely mapped at base pair resolution. These tools can be used to construct precise deletions in a homogeneous genetic background, for example, facilitating the analysis of complex regulatory regions [31], or genome-wide analysis of gene dosage effects to provide insights into gene regulatory network robustness [32]. Finally, *FRT* elements may be combined to generate other types of chromosomal aberrations, including translocations and inversions [30], and we have used such an approach to examine the consequences of disrupting developmental gene regulatory neighbourhoods [33]. The ability to precisely manipulate genomes at the chromosome level remains a powerful tool in the research armoury and the Bloomington Deficiency kit, rebuilt using the Flp-*FRT* approach, remains a widely used resource for the type of gene mapping and pathway expansion studies that have been important in building developmental networks [34].

The phiC31 system is a particularly useful tool for generating transgenic flies since a range of different transgenes may be inserted at an identical site in the genome [17,35], ameliorating concerns about position effects or other insertion site artefacts when comparing the effects of different constructs. PhiC31 integrase is directional and highly specific, mediating recombination between *attP* and *attB* sites to generate hybrid *attL* and *attR* sites that are not substrates for the enzyme (Figure 1d). A set of lines containing landing sites across the genome are available from stock centres and at least some of these have been functionally characterised in terms of expression properties [36]. Recently, a mutated integrase has been developed that allows reversal of the integration reaction [37], which is useful for building arrays of different transgenes at single sites in the genome. A second very useful approach available with phiC31 is recombinase-mediated cassette exchange (RMCE) [38]: in this case a genomic landing site containing a marker gene flanked by *attP* sites may be replaced by any other DNA sequence via a plasmid containing a gene or sequence of interest flanked by *attB* sites (Figure 1e). The RMCE system is incredibly powerful and since its introduction has been deployed as a component in a wide variety of genome engineering strategies (i.e., see Minos-mediated integration cassette (MiMIC) below).

**Figure 2:**
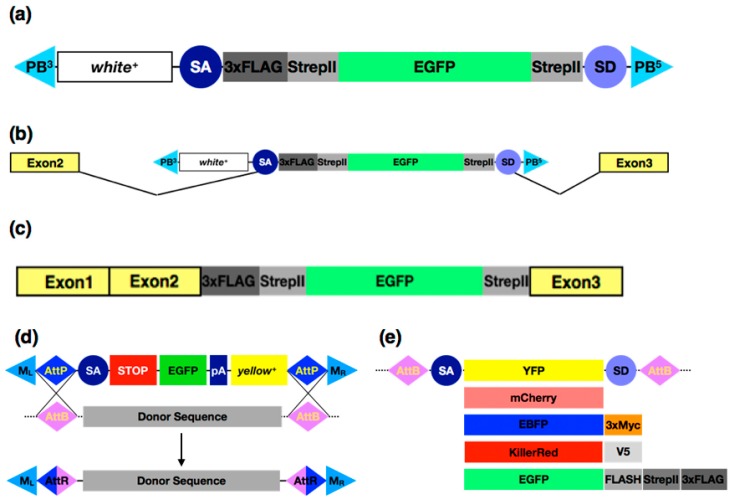
**Protein Trapping.** (**a**–**c**) ***piggyBac* transposon-based protein trapping**. (**a**) The *pigP* protein trap element used in [47]: the transposon ends (blue triangles) flank a genetic marker gene (*white*) and an artificial exon, which in this case contains the coding sequence for enhanced green fluorescent protein (EGFP) along with StrepII and 3XFLAG tags between splice donor and splice acceptor sites (blue circles). (**b**) Insertion of the transposon into an intron of a protein coding gene (represented by lines separating the yellow boxes) allows the possibility of splicing the artificial exon into the gene transcript. (**c**) If the transcript carrying the artificial exon is translated a tagged protein is generated. (**d**,**e**) **The** Minos-mediated integration cassette **(MiMIC) System.** (**d**) A *Minos*-based transposon with transposable element (TE) ends indicated by blue triangles, contains two *attP* sites in inverted orientation (blue diamonds) flanking a gene trap cassette with a splice acceptor (blue circle), stop codons in all three reading frames (red box), a fluorescent marker (EGFP) followed by a polyadenylation signal (blue box) and a genetic marker (*yellow*)*.* The sequences internal to the *attP* sites may be replaced via a RMCE reaction by providing a donor sequence flanked by *attB* sites and a source of phiC31 integrase. (**e**) A variety of different fluorescent reporters have been developed that can be used to introduce tags into genes with MiMIC insertions in coding introns via RMCE.

A key requirement for understanding gene function during development is the ability to localise gene products in space and time at the organismal, cellular and subcellular levels. For many years immunohistochemistry or immunofluorescence using specific antibodies was the primary method for collecting these data. Where antibodies are not available or there is a requirement for live imaging, transgenes containing tagged versions of a protein of interest have been useful. It is most common to express genes encoding tagged proteins under the control of the Gal4-*UAS* system, however, there can be concerns that over or misexpression may affect function. While it is sometimes possible to generate tagged transgenes that rescue null mutants, endogenous regulatory sequences are often too large or poorly characterised to make such an approach generally applicable. To try and address this P[acman] bacterial artificial chromosome (BAC) libraries for *D. melanogaster* [39] and Fosmid libraries for both *D. melanogaster* and *D. pseudoobscura* have been generated, which allow recombineering approaches in *E. coli* to generate tagged transgenes that are then integrated into the fly genome [40,41]. It would however clearly be preferable to tag endogenous loci in situ. Two transposon based approaches have been developed to facilitate in vivo protein tagging: the MiMIC method described below and various protein trap strategies. In the latter, a transposon containing an artificial exon, usually a fluorescent protein flanked by splice acceptor and donor sequences, is mobilised in the germline and progeny inheriting fluorescent reporter expression are recovered. A comprehensive screen requires a set of three vectors, each with the reporter in a different open reading frame. When the transposon inserts into an intron of a protein coding gene in the correct orientation, splicing will incorporate the artificial exon into the primary transcript and hence the fluorescent reporter into the protein [42] (Figure 2a–c). Due to the bias observed with *P* elements, particularly insertion hotspots in promoter and 5′ UTR regions [43], the use of *piggyback*-based transposons was found to generate a much higher frequency of bona fide protein traps. Together, three large-scale studies screened approximately 150 million embryos, recovering a little over 600 verified protein traps between them. Reassuringly, the majority of protein traps do not appear to substantially affect protein function [44,45,46]. While this may seem a relatively poor yield for such an effort, the lines generated have been incredibly useful for imaging studies down to the subcellular level [47] and have also facilitated mass spectrometry-based analysis of in vivo protein complexes that are purified via the tag [48]. Providing the ability to characterise protein localisation and interactions, protein trap lines have been used to study diverse developmental processes, including live imaging of anterior-posterior (A-P) polarity formation during oogenesis, the cell biology of epithelial morphogenesis and signalling pathway interactions [49,50,51]. Trapped lines have also facilitated the characterisation of genome wide transcription factor binding via chromatin immunoprecipitation (ChIP)-based studies [52].

One of the most useful and flexible transposon-based strategies available to the fly biologist is the MiMIC system [53] (Minos-mediated integration cassette): a collection of over 7000 *Minos*-based insertions that provide a platform for a variety of targeted engineering approaches. The core MiMIC transposon contains inverted phiC31 *attP* target sites within the *Minos* ends, a splice acceptor site followed by stop codons in all three reading frames. The element also contains a transcriptional/translational cassette encoding EGFP followed by a polyadenylation signal and a phenotypic marker (*yellow*). Insertion of a MiMIC element in the correct orientation into an intron of a coding gene will generate a truncated protein due to the splice acceptor and stop codons, thus acting as a gene trap. The true utility of MiMIC is the ability to use the phiC31 *attP* sites to replace the contents of the transposon with any piece of DNA via the RMCE system (Figure 2d). For example, regulatory sequences may be added to genes, functional reporters such as Gal4 or Flp may be inserted within 5′ UTRs or, in cases when inserts are within coding introns, a wide range of protein tags may be introduced. Around 30 different cassettes have been generated by the MiMIC team (Figure 2e), but any desired sequence can be easily cloned and introduced into the genome using the system. More recently, the team developed a genetic approach to circumvent the requirement for embryo injection [54], speeding up the generation of cassette replacement. Approximately 2000 genes currently have MiMIC insertions within introns, but the possibility of using CRISPR (see below) to place MiMIC insertions into the genome opens up the possibility that every intron containing gene in the genome could be amendable to protein trapping. The utility of the MiMIC system is attested to by over 100 research papers citing its use in a little over six years.

Whether protein trap or engineered MiMIC strategies are used to generate tagged proteins, there are some useful approaches to knock down their function [55] in a tissue or temporally specific manner. Transcripts containing inserted tags may be targeted by RNA-mediated interference (RNAi) constructs directed against the tag, and lines containing *UAS*-driven RNAi recognising green-fluorescent protein/enhanced green fluorescent protein (GFP/EGFP) are available from stock centres. A second approach is via the use of an anti-GFP nanobody, which targets GFP-tagged proteins for degradation via the ubiquitin pathway [56,57] and *UAS*-driven lines are available. Both these methods have been shown to be effective with in vivo tagged proteins and offer a level of functional control over gene products that can, in principle, be monitored in real time via imaging of the fluorescent tag. However, despite the relative ease and simplicity of these methods they have obvious limitations; the elimination of tagged proteins is relatively slow and they are not readily reversible. For the rapid elimination of gene function, an auxin-inducible degradation system (AID) has recently been shown to work in *Drosophila* [58]. This system offers the ability to regulate the inducible and rapid degradation of specific target proteins by the recruitment of proteins with an auxin-inducible degron to the E3 ubiquitin ligase complex. A disadvantage of this system is the need to introduce a specific tag containing an auxin-inducible degron domain into the gene of interest, though this could be achieved for genes harbouring an appropriate MiMIC insertion.

## 3. CRISPR/Cas9

### 3.1. Overview

As the above discussion emphasises, the range of transposon based tools available to the *Drosophila* biologist is impressive, however, even here the landscape for genome engineering has changed dramatically with the introduction of the CRISPR-Cas9 system. Clustered, Regularly Interspaced, Short Palindromic Repeat (CRISPR)-based engineering has become the most popular and commonly used tool for genome manipulation over the last five years. Since its discovery, or rather understanding its potential, numerous studies have confirmed the wide applicability and adaptability of the system [59,60,61] and a number of different methods have been developed with different variants of the RNA-guided Cas9 endonuclease adopted for genome editing [62]. 

**Figure 3:**
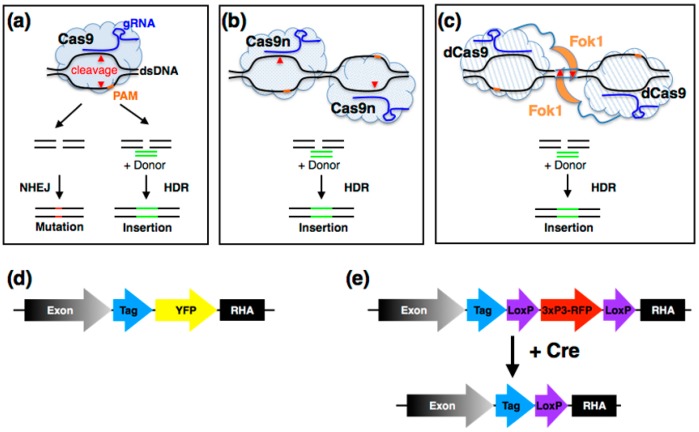
**CRISPR Genome Engineering.** (**a**) The wild-type Cas9 complex (light blue cloud) contains the Cas9 endonuclease and a guide RNA (gRNA) (blue) complementary to the target site adjacent to a PAM sequence (orange). The complex opens the DNA duplex and introduces a double strand break (red triangles). Repair by the non-homologous end joining (NHEJ) pathway may result in indel mutations whereas homology directed repair (HDR) in the presences of a donor template (green) generates insertions. (**b**) Using mutant Cas9n enzymes that make single strand cuts with two gRNAs (blue) direct the Cas9n complexes to make cuts (red triangles) separated by some distance. The gap may be repaired in the presence of a donor (green) to generate an insertion. (**c**) Dead Cas9 (dCas9) enzymes (which are unable to cleave DNA) are fused with FokI nuclease monomers (orange). When two gRNAs (blue) some distance apart are used, dCas9-FolkI monomers are brought into proximity allowing the FokI to dimerise and cleave in between. The resulting gap may be repaired in the presence of a donor (green) to generate an insertion. (**d**) Diagram of donor cassette for direct addition of a protein tag to the C-terminal of a coding exon. The donor DNA contains part of the exon sequence with a biochemistry tag and yellow fluorescent protein (YFP) in frame, followed by a right hand end homology arm (RHA) that can mediate the type of insertion event shown in (**a**). (**e**) Diagram of a donor sequence used to introduce biochemistry tags to the C-terminal of a coding exon along with a removable marker. The donor DNA contains part of the exon sequence with a biochemistry tag in frame. This is followed by an eye expressed red fluorescent protein (RFP) cassette flanked by *LoxP* sites, which are downstream of the splice donor site and thus within an intron, then a right hand homology arm. Once a verified insertion has been recovered, tracked by the acquisition of RFP, the RFP is removed by exposing to Cre recombinase and recovering flies who have lost RFP expression. The resulting lines have the tagged exon and a single intronic *LoxP* site.

CRISPR-Cas systems are natural RNA-guided adaptive immune systems that provide protection mechanisms against viruses or other exogenous DNA entering bacterial and archaeal cells. The most studied and widely applied system for genome editing, Class 2 CRISPR-Cas from *Streptococcus pyogenes*, uses a guide RNA (gRNA) composed of a specific CRISPR RNA (crRNA) combined with a tracrRNA sequence to guide the Cas9 nuclease to a genomic target site where it introduces a double-strand break (DSB) that is repaired by endogenous repair pathways. The system requires only 20 nucleotides of homology to the target site in the gRNA, but the target must be adjacent to a three nucleotide (NGG) protospacer adjacent motif (PAM) in the genome (Figure 3a) [63,64]. Repair may be via the non-homologous end joining (NHEJ) pathway [65], leading to insertions and/or deletions (indels) at the target site. Alternatively, the activity of homology-directed repair (HDR) pathways can facilitate precise genome editing by introducing alternative sequences via a supplied donor DNA (Figure 3a) [66,67]. While the initial efficiency of specific targeting, especially when trying to introduce mutations or tags, was initially low and variable [68,69], various improved systems have been developed. The method originally relied on delivery of the nuclease and RNA components via microinjection of RNA or DNA, the development of transgenic lines expressing Cas9 under the control of germline promoters such as *nanos* (*nos*) or *vasa* as recipients for injection of plasmids expressing gRNAs from U6 promoters has considerably simplified the process and can generate desired events in the genome with higher efficiency than with the initial injection approach [70].

One of the major concerns in any genome engineering work, whether using CRISPR/Cas9 or any other system, is the possibility of off-target effects resulting from gRNA binding to imperfectly matched targets at other genomic locations [71]. While the extent of off-target effects has not been fully characterised, several studies that suggest that for relatively small genomes, such as that of *Drosophila*, off-targets may be less of an issue compared to organisms with larger genomes [72,73]. The problem may be, at least in part, overcome by using a mutant version of Cas9 developed by Cong and colleagues [74], Cas9D10A (Cas9n), that makes single strand nicks at the target locus [74,75,76]. When used in conjunction with two gRNAs flanking the desired target site and a donor repair template, repair is generally via the high-fidelity HDR pathway [69,77]. The nickase activity of Cas9n does not activate the NHEJ pathway and while Cas9n is certainly more specific than wild-type Cas9, DSBs are still detectable at target sites when only one gRNA is used and it is possible that Cas9n may cause indels at off-target sites due to either of the gRNAs. Encouragingly, the paired Cas9n system has been used to generate large deletions without observing unwanted translocations, and so forth (Figure 3b) [76,78], and a recent comparative study indicates that Cas9n can induce more HDR than NHEJ, at least in human HEK293 cells [79].

Concerns regarding the use of Cas9n have been raised due to the possibility of DNA rearrangements via single nicks [62]. These unwanted off-targets can result from the recognition of multiple genomic sites by gRNAs that that can subsequently be cleaved. In contrast to other nuclease systems for genome engineering, such as zinc finger nucleases or transcription activator-like effector nucleases (TALENs), that rely on dimerization for target cleavage, Cas9 acts as a monomer. In order to increase specificity, new Cas9 versions that only cleave when dimerised have been developed: for example, a nuclease dead Cas9 (dCas9) fused to the non-specific endonuclease FokI has the gRNA directed specificity of the CRISPR system but relies on obligate FokI dimerization for cleavage (Figure 3c) [63,64,80,81]. A clear limitation of the paired dCas9-FolkI approach is the requirement for two appropriately spaced gRNA sites 15–25 bases apart [81]. This constrains the widest applicability of the method since these conditions cannot always be achieved. Alternatively, there have been efforts to develop engineered Cas9 enzymes (i.e., SpCas9-HF1) with increased fidelity to ensure more specific targeting [82], however, there are reports of lower efficiencies with such variants [83]. Irrespective of the engineering method used, effects of off-target events may be largely ameliorated by cleaning up stocks via standard back-crossing methods.

The obvious restriction when using Cas9 for genome engineering is the requirement for a PAM sequence adjacent to the desired target site, which limits the number of Cas9 engineerable sites in a genome. To overcome this limitation several mutant versions of Cas9 with altered specificities have been engineered [84] and Cas9 homologs from other organisms that show different PAM specificities have been isolated. For example, a recently identified class 2 endonuclease, Cpf1 from *Francisella novicida*, has received some attention [85]. The Cpf1 PAM is TTN and is located 5′ to the target site (in contrast to the Cas9 PAM which is 3′ to the target), providing a new set of potential targets not accessible to Cas9 systems. Finally, other Class 2 CRISPR effectors, such as Cas13a (C2C2), have been shown to target RNA, however, few studies have explored the potential of this activity [62,86]. It should be remembered that the potential of CRISPR based systems has only been apparent for five years and the progress in developing variants systems with different properties has been tremendous and will surely continue to provide new enzymes and systems with improved specificity and fidelity.

### 3.2. Mutagenesis with CRISPR/Cas9 Systems

The most straight forward way to modify the genome using CRISPR/Cas9 based genome engineering is the introduction of short insertions/deletions (indels) by inducing NHEJ, which frequently leads to frameshift mutations and thus null alleles or truncations of the targeted gene product. All that is required is the Cas9 protein and a single gRNA targeting the gene of interest after the transcription start site [87]. Large deletions of 30 Mb have been generated in human cell lines and even chromosomal rearrangements such as translocations can be generated by providing two gRNAs, targeting either side of the region to be deleted or sites on two different chromosomes [87,88,89]. Insertions of up to 5.7 kb have been achieved by providing linear DNA during CRISPR/Cas9 induced NHEJ in Zebrafish [90], and up to 34 kb in human cell lines [91] using *piggyBac* or adenoviral vectors that were cleaved by Cas9, targeted via a vector specific gRNA. A drawback is that the position of the knock-in is not precisely defined, and thus a molecular screen for in-frame insertions must be performed. It remains to be determined to what extent NHEJ-driven rearrangement can be exploited in *Drosophila*.

The considerable versatility of CRISPR/Cas9 based genome editing lies in the utilization of homology directed repair (HDR) for the precise incorporation of a donor sequence in the form of ssDNA-oligonucleotides, PCR (Polymerase Chain Reaction) products or plasmids, that are carried between homology arms, which are used as templates during the double strand repair process rather than the homologous chromosome [69,92]. Depending on the target site(s), the gRNA(s), the homology arms used and the region between them, different outcomes are possible. An insertion is achieved when the donor carries a sequence between the homology arms that cover the regions directly adjacent to the double strand break, thus inserting the sequence (Figure 3a). In this way, for example, additional amino acids can be added to a coding exon, point mutations introduced, or new binding sites added to a regulatory region. The optimum length for homology arms appears to be approximately 1 kb [93]. If the homology arms are separated with respect to the target genomic DNA the donor DNA between the homology arms may replace the intervening genomic segment. Since HDR efficiency decreases with the distance of homology deviations from the double strand break, replacement or deletion of large regions can be obtained by providing two gRNAs, each next to a homology arm, thus actively excising the intervening region [93,94,95]. Again, the sequence between the homology arms is inserted. Note that if homologous recombination does not take place the region between the homology arms may still be deleted, which obviously generates a deletion and perhaps a null allele. While a null allele can also be produced when only one gRNA is used as indicated above, this is likely to generate a frame shift mutation, which may be less useful, especially in non-coding regions. If a specific point mutation at a locus is desirable, a dCas9-cytidine deaminase chimeric enzyme may be used rather than homology directed replacement. The deaminase catalyses the conversion of C to U residues thus generating C to T or G to A substitutions. This ‘base editing’ is generally more efficient than HDR-mediated point mutation and at the same time minimizes indel formation [96]. This has recently been complemented by conversion of A-T to G-C base pairs by using a transfer RNA specific adenosine deaminase fused to catalytically impaired CRISPR/Cas9 [97].

Initial screening for positive events can be done using standard PCR, high resolution melt analysis (HRM), or sometimes more easily with visual markers. These can include body or eye colour markers, or fluorescent proteins expressed, for example, in the eye via the 3XP3 promoter (Figure 3e). Such markers are usually flanked by *FRT* sites for subsequent removal to avoid interference with gene function or other unphysiological responses [98]. Whichever method is used, any mutations should be verified by sequencing. Experiments ranging from the generation of specific single base pair mutations to replacement of whole genes have been described [99,100,101]. The latter is especially relevant for evo-devo studies, for example, to define the degree of functional redundancy between paralogous or orthologous genes by swapping the coding sequences of one gene into the genomic regulatory context of another. An example of such an approach is a swap of *Sox3* with *Sox2* coding sequences in mouse, facilitated by the simple genomic architecture of this class of genes since they only contain a single exon [102]. We have recently used a similar approach with the fly orthologues of these genes (S. A. Koestler and S. Russell, unpublished). Such experiments become more complex for larger genes, which may consist of several exons separated by introns, especially if the donor or host genes have intronic regulatory sequences. In such cases addition of a cDNA to functionally replace a null allele at a donor locus may be considered, although it is probably prudent to first test whether the coding sequence of the host gene is sufficient to effect rescue. For example, using this approach, gene and domain swaps in *D. melanogaster* as well as between *D. melanogaster* and *Tribolium castaneum* have led to the dissection of the regulatory function and evolution of members of the *robo* gene family [103,104]. However, fully understanding redundancy requires the removal of all redundant members of a gene family [105]. While such efforts previously required crossing or recombination of single mutants, or removal of large chromosomal regions [106,107], CRISPR/Cas9 allows a much more precise and efficient approach. Encouragingly, as the requirement for ever more high-throughput analysis is increasing, up to four genes have already been targeted in parallel by multiplexing [108,109].

The major challenges currently encountered when applying CRISPR/Cas systems for generating mutations arise from the relatively low efficiency of the desired genome modifications and the lack of an obvious phenotype in heterozygous F1 progeny, hence the need for time-consuming molecular screening of positive variants mentioned above. Due to the laborious nature of such screens and the need to examine many progeny, several groups developed methods facilitating more efficient engineering and hence more rapid screening. Bullock and colleagues [70] developed a versatile toolbox for *Drosophila* genome engineering consisting of a set of evaluated transgenic Cas9 lines and gRNA-expression plasmids. Their studies indicated that injecting gRNA(s) into Cas9 transgenic flies increases the overall efficiency but a fully transgenic system where the gRNA expressing construct is also inserted in the genome results in much higher efficiency. In principle, this strategy requires the generation of a transgenic fly line for every gRNA of interest that subsequently needs to be crossed to a transgenic Cas9 line. Despite being more time consuming initially, the fully transgenic system means that all transgenic flies pass on mutant alleles to their progeny, with efficiencies approaching 100% observed with the majority of tested gRNAs. Consequently, there is a significant improvement in screening efficiency since the majority of progeny have an engineered chromosome. More recently, based on the fact that that multiple CRISPR events can occur in a single cell [74,110], the co-CRISPR or co-conversion method initially developed in *C. elegans* [111,112] has been also successfully applied in *Drosophila* [113]. The method relies on simultaneously targeting a gene of interest and an *ebony* marker gene: *nos-Cas9* embryos [70] are injected with a mix of gRNAs targeting both *ebony* and the gene of interest (with or without a donor template). It is expected that in any given cell where *ebony* is mutated it is likely that the Cas9 has also been active at the gene of interest, thus progeny showing loss of *ebony* are selected for molecular analysis of the target gene [113]. In *Drosophila*, the co-CRSIPR strategy was found to be more efficient for identifying NHEJ mutagenesis events rather than HDR knock-in events. A similar approach targeting *white* as an editing marker has also been reported [114], however, it should be noted that *white* is a much more common transgenesis marker than *ebony* and thus may preclude its use in some crossing schemes. These efficiency and marker gene approaches simplify the task of screening for desired engineering events by enriching for successfully edited progeny and provide an excellent framework for further development of strategies to rapidly isolate mutations.

The generation of stable stocks carrying defined gene modifications is most often achieved with a germline expressed Cas9, usually via the *vasa* or *nanos* promoters described above [70,90,115,116]. However, in some situations modification of the germline may be either lethal or undesirable and it is therefore necessary to generate mosaic animals. These can be made by simply injecting the gRNA and donor construct (if one is used) into embryos ubiquitously expressing Cas9 or alternatively by co-injecting a source of Cas9. For the detection of successfully modified cells in mosaics the use of a marker is strongly recommended. The marker can be a fluorescent protein under control of a ubiquitous promoter carried on the injected donor construct. Although using a marker greatly facilitates the screening process it is not always possible to apply and the other approaches described in the previous section need to be employed. An alternative is to utilise the Gal4/*UAS* system to drive Cas9/gRNA expression in a specific cell type or at a particular developmental stage. Conditional mutants can be established, but the time it takes until they take affect and the turnover rates of the involved agents have to be considered [69,117]. It must be emphasized that this approach has to be used with care due to complications arising from the possible introduction of multiple mutations and cellular heterogeneity, especially when NHEJ is the desired repair event or gRNAs are not highly specific. Additional control and flexibility in the design of experiments can be obtained by combining CRISPR/Cas9 with other genome engineering techniques, for example, by insertion of *FRT* or *attP* sites [69]. Zhang et al. took this a step further by combining CRISPR/Cas9 with RMCE [118] to facilitate the analysis of several different constructs at the same locus.

A given gene product often exerts its function in a variety of different cellular contexts, that is, a transcription factor used in different cells during development, and this is reflected in the control of its expression by a combination of multiple regulatory sequences [119,120]. To probe these non-coding, regulatory regions the same CRISPR/Cas9 approaches that are being applied to coding sequences are useful. These include deletion [121], insertion or swapping of regulatory elements to study functional redundancy, for example, of distributed (primary and shadow) enhancers [122,123,124]. Single base pair changes are suited to determine the relevance of specific positions in transcription factor binding motifs or to alter splice sites [125,126]. The readout, however, is distinct. Regulatory sequences and their combinatorial logic can be revealed by examination of expression patterns, transcription factor binding, their DNA binding dynamics, and chromatin state or three dimensional nuclear chromatin organization [88,127,128]. Knowledge of regulatory modules also allows generation of more refined tools by driving cell type and developmental stage specific expression of CRISPR/Cas9 components [123,129]. Databases such as the super enhancer archive (http://sea.edbc.org) [130] that integrate results from various types of experiments and CRISPR/Cas9 target sites can aid in the design of experiments. Integration of all these data with fluorescent tagging of gene products at their endogenous loci (see below) will facilitate measurement of local concentrations. Together, this will significantly contribute to a more comprehensive understanding of gene regulation, which will culminate in more precise mathematical models [120]. 

### 3.3. Protein Tagging with CRISPR/Cas9 Systems

As with transposon based approaches, the use of CRISPR/Cas9 genome engineering for protein tagging is beginning to provide new tools for developmental biology. In contrast to the methods described above, the CRISPR system facilities the precise introduction of protein tag sequences at endogenous loci [70]. Thus, tagged proteins are expressed under the control of endogenous regulatory sequences, more likely to be expressed at physiological levels and, if relevant information is available, may be engineered to minimise the impact on normal protein function. Introduced protein tags may be visible fluorescent proteins, for example, YFP, GFP, mCherry, and so forth [131] (Figure 3d) or short epitope tags (FLAG, STREPII, Myc, etc.) that, due to their small size, are less likely to perturb protein function. Tagged proteins may be visualised in vivo via fluorescence microscopy or immunohistochemistry and tags may also be used in biochemical studies, for example complex purification combined with mass spectrometry [48,132].

Due to the fact that the small size of epitope tags is likely to reduce perturbation since they can be more readily incorporated into a protein scaffold, versatile protein tagging approaches with split fluorescent proteins have been developed [133]. Such self-complementing split fluorescent proteins enable live cell imaging when they are used as epitope tags and have been shown to work in *Drosophila* cells [134]. The other advantage, aside from the small tag size, is the ease with which it is adaptable to genome engineering since successful tag knock-in via CRISPR-mediated HDR can be achieved with readily synthesised single stranded DNAs of ~200 nucleotides that include the tag and sufficient homology arms to mediate recombination. Alternatively, the tags can be introduced with removable markers that provide convenient reporters for successful recombination and subsequent genetic crossing. These can be readily removed from the genome via one of recombination systems (e.g., Cre-*Lox* or Flp-*FRT*) described above, leaving only the tag of interest and a short *LoxP* or *FRT* site [69,135] (Figure 3e). The development of ‘scarless’ techniques can further limit the introduction of unwanted additional sequences into the genome [98]. A method combining both CRISPR and *piggyBac* transposase utilises the pHD-ScarlessDsRed vector, available from the *Drosophila* Genomics Resource Center (http://flycrispr.molbio.wisc.edu/scarless). Another possibility is that *LoxP* or *FRT* sites could be replaced by a gRNA to efficiently mediate the marker removal via CRISPR, however, a limitation of this approach is that NHEJ events could lead to the recovery of INDELs.

The use of CRISPR/Cas9 for tagging proteins in situ is becoming widespread and opens up genome biology approaches to virtually any desired protein in the genome: for example, ChIP or RNA immunoprecipitation (RiP) methods for identifying the binding targets of DNA or RNA binding proteins respectively, rely on the availability of highly specific antibodies. Even when good antibodies are available, it is often difficult to make accurate comparisons between samples because different antibodies do not behave in the same way. The possibility of performing such experiments for multiple different proteins with a single antibody is clearly attractive and an obvious route to achieving this is by the introduction of in situ epitope tags. Multiply RNA binding proteins have been engineered with V5 and FLAG tags [136]. Similarly, methods for introducing FLAG (CETCh-seq) [137] or FLAG and fluorescence tags (cmChIP-Seq) [138] have also been developed recently [138]. The latter combines CRISPR with microhomology mediated end joining (MMEJ) to tag transcription factors and, while it has currently been shown to be successful in human cell lines, MMEJ is certainly active in *Drosophila* [139]. 

Koles et al. [140] developed a strategy to enable tissue specific tagging of endogenous loci in *Drosophila* (T-STEP) by combining CRISPR-Cas9 with a yeast recombinase (Rippase). In brief, a knock-in cassette comprising tandem Rippase-specific Recognition Sequences (RRS) in frame with the targeted protein brackets a strong lethality selection marker (Golic+ [141]) to ensure high efficiency targeting: an in frame GFP is 3′ to the RRS site. Tissue specific expression of the Rippase via the Gal4-*UAS* system allows removal of the T-STEP cassette and puts the GFP in frame with the protein. The authors used this approach for live imaging of endosomal components. In cultured cells, Kunzelmann et al. [142] recently described a simplified protocol for rapid genome engineering that indicates homology arms as short as 60 bp are sufficient for efficient HDR. Their work provides a number of improvements to the system in terms of gRNA design and expression, making routine tagging of proteins in cultured cells feasible. Interesting, these authors more recently showed that the presence of selection markers in vectors for engineering cell lines induced siRNA-mediated changes in gene expression and thus removal of the marker when using such approaches [143] is desirable. 

While specific reports of in vivo tagging in *Drosophila* are currently limited, anecdotally it is clear that many laboratories are actively using the technology and it is obvious that CRISPR mediated strategies will be widely applicable for tagging protein isoforms. For example, we have recently used fluorescent or small epitope tags to specifically tag different isoforms of *shaggy*, *ventral nervous system defective* and *roadkill* amongst others, using both direct tagging and Cre-*Lox* directed marker removal, with high efficiency (D. Korona and S. Russell, unpublished).

## 4. Conclusions

Taken together, the methods we have described here are increasing the utility of the already well-established *Drosophila* model for exploring developmental processes. From the generation of specific mutations through to the ability to track proteins in real time in vivo, genome engineering is opening new possibilities for characterising the deployment of the genome: from the characterisation of gene regulatory networks through post-transcriptional gene control, to the cell biology of proteins and protein complexes. These developments will continue to keep the fly at the forefront as an experimentally tractable system for understanding metazoan biology.
